# Short-Term Effects of Extreme Heat, Cold, and Air Pollution Episodes on Excess Mortality in Luxembourg

**DOI:** 10.3390/ijerph22030376

**Published:** 2025-03-04

**Authors:** Jérôme Weiss

**Affiliations:** Epidemiology and Statistics Unit, Health Directorate, Ministry of Health and Social Security, L-1433 Luxembourg, Luxembourg; jerome.weiss@ms.etat.lu

**Keywords:** excess mortality, environmental risks, distributional regression, attributable mortality, extreme events

## Abstract

This study aims to assess the short-term effects of extreme heat, cold, and air pollution episodes on excess mortality from natural causes in Luxembourg over 1998–2023. Using a high-resolution dataset from downscaled and bias-corrected temperature (ERA5) and air pollutant concentrations (EMEP MSC-W), weekly mortality p-scores were linked to environmental episodes. A distributional regression approach using a logistic distribution was applied to model the influence of environmental risks, capturing both central trends and extreme values of excess mortality. Results indicate that extreme heat, cold, and fine particulate matter (PM_2.5_) episodes significantly drive excess mortality. The estimated attributable age-standardized mortality rates are 2.8 deaths per 100,000/year for extreme heat (95% CI: [1.8, 3.8]), 1.1 for extreme cold (95% CI: [0.4, 1.8]), and 6.3 for PM_2.5_ episodes (95% CI: [2.3, 10.3]). PM_2.5_-related deaths have declined over time due to the reduced frequency of pollution episodes. The odds of extreme excess mortality increase by 1.93 times (95% CI: [1.52, 2.66]) per extreme heat day, 3.49 times (95% CI: [1.77, 7.56]) per extreme cold day, and 1.11 times (95% CI: [1.04, 1.19]) per PM_2.5_ episode day. Indicators such as return levels and periods contextualize extreme mortality events, such as the p-scores observed during the 2003 heatwave and COVID-19 pandemic. These findings can guide public health emergency preparedness and underscore the potential of distributional modeling in assessing mortality risks associated with environmental exposures.

## 1. Introduction

Environmental risks have a well-established influence on public health, with strong evidence linking extreme temperatures and air pollution to increased mortality [1]. Heatwaves are consistently associated with elevated mortality rates across regions [2,3,4], while cold spells similarly increase mortality risks [5,6]. Additionally, air pollutants such as particulate matter (PM_10_, PM_2.5_), ozone (O_3_), and nitrogen dioxide (NO_2_) are tied to higher mortality from respiratory and cardiovascular diseases [7,8,9,10]. While the relationship between environmental risks and mortality is well-documented globally, no study, to our knowledge, has specifically quantified these associations for Luxembourg. Most insights come from larger multinational studies [11,12], which may not fully reflect the country’s specific environmental and demographic characteristics. Addressing this gap is essential for developing tailored public health interventions.

National exposure to environmental risks for the general population is frequently estimated using monitoring station data [13]. However, these stations often capture localized conditions that may not accurately represent broader exposure, especially when located away from densely populated areas [14]. Complementary to station observations, global reanalyses provide time series of continuous spatial data, enabling more consistent and representative exposure assessments across the general population [15].

In epidemiological, statistical analyses, the focus is often on understanding the average behavior of health variables. While traditional methods concentrate on average mortality effects, distributional regression models offer a broader approach by modeling not just the mean but also other distributional parameters, such as variance and skewness [16,17]. This allows for a more comprehensive understanding of how covariates influence the entire conditional response distribution. Extending the analysis beyond the conditional mean also provides a better understanding of extreme events, which, although rare, can have significant public health consequences, particularly when the healthcare system is overwhelmed by peaks in mortality. Traditional statistical models, which focus on average or typical events, may fail to capture the occurrence and intensity of these events, which could lead to inadequate predictions and decision-making [18,19]. However, methodologies dedicated to extremes remain scarce in epidemiological research. A warning system based on extreme value theory (EVT) for detecting outbreaks was developed by [20], while EVT was also used by [21] to predict extreme influenza mortality events. A model for hospitalizations and death peaks due to cardiovascular diseases in Quebec was proposed by [22]. In a later study, ref. [23] applied quantile regression to examine the relationship between cardiovascular health peaks and meteorological factors in Montreal. Extremes in systolic blood pressure were modeled by [24], while [25] investigated the influence of meteorological extreme conditions on flu-related hospital visits. While prior studies primarily modeled only the intensity of extreme health events, this study also investigates the factors driving their occurrence. This dual focus offers a more comprehensive understanding of the environmental conditions that contribute to excess mortality.

This paper aims to evaluate the short-term effects of heat waves, cold waves, and air pollution episodes (PM_2.5_, O_3_, and NO_2_) on excess mortality from natural causes (i.e., deaths caused solely by disease or natural processes) in Luxembourg. Leveraging a high-resolution dataset spanning 1998–2023, the analysis employs distributional regression modeling to quantify the impact of these environmental factors on excess mortality, focusing on both the occurrence and intensity of extreme mortality events. Findings are intended to inform public health strategies in Luxembourg to effectively mitigate these environmental risks.

## 2. Materials and Methods

### 2.1. Data Sources

The mortality dataset used in this study was drawn from Luxembourg’s official causes-of-death register sourced from death certificates managed by the Epidemiology and Statistics unit of the Luxembourg Health Directorate [26]. Data are available from 1998 to 2023. The register is exhaustive and includes data on all deaths occurring on Luxembourg territory (residents and non-residents). Causes of death were coded according to the ICD-10 classification system [27]. The analysis focused on deaths from natural causes among residents only, excluding stillbirths. Natural causes were defined as deaths classified under ICD-10 codes A00–R99, thereby excluding external causes of death. Weekly age-standardized mortality rates (ASMRs) from natural causes were calculated for the period 1998–2023. Annual population data were obtained from the National Institute of Statistics and Economic Studies of Luxembourg (STATEC) and linearly interpolated to a weekly resolution. Mortality rates were age-standardized using the latest available 2013 European Standard Population to ensure comparability over time and between regions [28].

Daily minimum, maximum, and mean temperatures were obtained from both the ERA5 numerical reanalysis dataset [29,30] (spatial resolution: 0.25°) and 48 meteorological stations in Luxembourg. This includes the MeteoLux Findel Airport station, which has been operational since 1947, and 47 stations from the agrometeorological measurement network, which have been operational since 2000 for the longest series.

For pollutant concentrations, we utilized the EMEP MSC-W numerical model (European Monitoring and Evaluation Programme Meteorological Synthesizing Centre-West, spatial resolution: 0.1°), which simulates three-dimensional distributions of air pollutants across Europe and has proven to be suitable for epidemiological studies [31,32]. Observational data were acquired using the saqgetr R package [33], which connects directly to the Air Quality e-Reporting platform of the European Environment Agency. This platform provides air quality measurements from 12 stations in Luxembourg, which have been operational since 2004, for the pollutants studied. For both simulations and observations, we retrieved 24 h averages for NO_2_ and PM_2.5_, as well as 1 h daily maximum concentrations for O_3_.

### 2.2. Identification of Environmental Episodes

To reflect the national exposure of the Luxembourg population to the studied environmental risks, we used retrospective numerical models simulating the environmental variables, followed by a bias correction procedure using measurements from monitoring networks. Using numerical models overcame the limitations of relying solely on monitoring stations, which often have uneven spatial coverage and may experience data gaps. Observational networks can also evolve over time, complicating the creation of a coherent national time series. However, despite their extensive spatial and temporal coverage, numerical simulations often require bias correction to align accurately with observational data. To address this, we applied the methodology developed by [34], which combines downscaling and bias correction. This method was adapted to our application through the following steps:

1. Data extraction: daily gridded data were retrieved from numerical models and station measurements for the period 1998–2023, covering the Grand Duchy of Luxembourg;
2. Downscaling: both raw simulations and station measurements were downscaled to a 1 km × 1 km grid using Inverse Distance-Weighted (IDW) interpolation with the R package *gstat*;
3. Bias correction: at each point of the 1 km grid, downscaled simulations were bias-corrected using downscaled observations as a reference. This was achieved using monthly Empirical Quantile Mapping (EQM) with the R package *qmap*, which aligns the statistical distribution of the simulations with that of the station measurements month by month.

**Figure 1:**
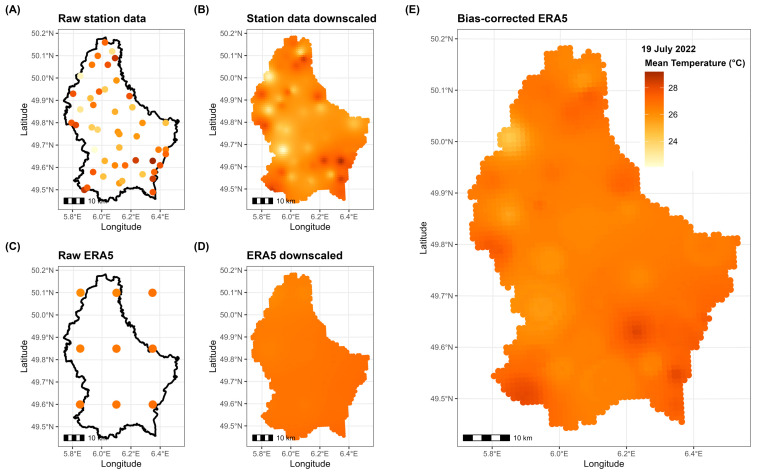
Workflow for downscaling and bias-correction of daily mean temperatures on 19 July 2022: (**A**) raw station data; (**B**) station data downscaled; (**C**) raw ERA5 simulations; (**D**) ERA5 simulations downscaled; (**E**) bias-corrected ERA5 simulations. Dots in panels (**A**,**C**) indicate station locations.

The same process was applied for both temperature and pollutant concentrations. Figure 1 outlines the workflow for daily mean temperatures on the heatwave day of 19 July 2022, from raw observations to the final bias-corrected model outputs.

Next, we calculated the daily national exposure series based on bias-corrected simulations for both temperatures and pollutant concentrations from 1998 to 2023. First, population-weighted daily averages were computed for each of Luxembourg’s 12 cantons, using population data from 2021 at a 1 km resolution as weights [35]. These cantonal series were then aggregated into a national daily average, with each canton weighted by its population for the corresponding year. This approach follows the methodology used in global burden of disease studies, where urban and rural PM_2.5_concentrations are population-weighted and aggregated at the national level [13]. By accounting for variations in population density, this method provides an accurate representation of national exposure [36,37].

Then, from the daily national exposure series, we identified environmental episodes as days of exceedances of the health-risk thresholds defined in Table 1. For temperature, extreme heat and cold days were classified according to Luxembourg’s red alert thresholds [38]: days with a maximum temperature of at least 35 °C and a previous day’s average temperature of at least 23 °C were categorized as extreme heat days, while days with minimum temperatures below −15 °C were designated as extreme cold days. Regarding air pollution episodes, thresholds were set according to the EU 2030 air quality standards aimed at protecting human health [39], aligning with World Health Organization recommendations [40]. These thresholds designate pollutant levels considered harmful to health: 25 µg/m^3^ for PM_2.5_ (24 h average), 50 µg/m^3^ for NO_2_ (24 h average), and 120 µg/m^3^ for O_3_ (8 h daily maximum). Since our ozone data represent the 1 h daily maximum, the 8 h threshold of 120 µg/m^3^ was converted to a 1 h equivalent of 160 µg/m^3^ using standard conversion ratios for ozone exposure time averages [41]. This 1 h threshold of 160 µg/m^3^ also aligns with the pre-information alert level currently used in Luxembourg. The use of absolute thresholds, rather than relative percentile-based definitions, was motivated by the policy-driven objective of this study, ensuring that the results could be directly applicable to national warning systems and regulatory frameworks.

### 2.3. Statistical Modeling of Excess Mortality

Similar to the approach of [42,43], we developed a statistical model to estimate excess mortality for the period 1998–2023. This involved calculating a baseline mortality, which was derived from weekly ASMRs and adjusted for confounding factors such as seasonality and long-term temporal trends. Instead of using a traditional mean regression approach based on an overdispersed Poisson model, we employed a median regression method, which yields greater robustness against the impact of extreme events, such as pandemics like COVID-19. This was implemented using the QGAM package in R [44]. Thin-plate splines were employed to account for long-term trends, while penalized cyclic cubic splines captured seasonal variations. From this baseline, weekly p-scores, indicating the percentage of deaths above expected levels, were computed to capture deviations from typical mortality patterns [45]. The use of age-standardized p-scores enables the implicit incorporation of structural population changes, unlike traditional p-scores [46,47]. Descriptive statistics, including the identification of weeks with the highest p-scores and the computation of Pearson’s correlation coefficient with death counts, were used to explore patterns related to the p-scores.

Then, we linked p-scores with weekly environmental episode counts for 1998–2023. To capture cumulative exposure effects, we summed the number of episode days across the current and preceding weeks. This approach accounts for episodes that occur late in the week and may influence the following week’s mortality. It also reflects various ways environmental episodes might impact mortality, capturing immediate, cumulative, and short-term delayed effects—an approach conceptually similar to distributed lag models frequently used in epidemiological studies [48].

We used a generalized additive model for location, scale, and shape (GAMLSS) to model weekly p-scores as a function of environmental covariates. We selected the logistic distribution for its symmetrical shape and heavier tails relative to the normal distribution, potentially capturing extreme values more effectively [49]. The cumulative distribution function of the logistic distribution is given by the following equation:

(1) $$\begin{array}{c} \;F\left(y\right)=P(Y<y)={\left[1+\exp\left(-\frac{y-\mu }{\sigma }\right)\right]}^{-1},\;y\;\in \;R\; \end{array}$$

where *μ* is a location parameter and *σ* a positive scale parameter. The distribution’s mean is *μ*, with variance of π^2^*σ*^2^/3. Both parameters, *μ* and *σ*, were indexed to each week *t* and modeled as linear functions of covariates. Specifically, these included the cumulative counts of environmental episode days (heat, cold, and pollution) over two weeks, along with control variables *p_flu_* and *p_COVID-19_*, which represent the percentage of weekly deaths caused by influenza and COVID-19, respectively. Additionally, we included an autoregressive term using the prior week’s p-score *Y*_*t*−1_ to account for potential short-term autocorrelation in weekly p-scores. For a given week *t*, the location and scale parameters *μ_t_* and *σ_t_* were thus specified as follows:

(2) $$\begin{array}{c} \;\left\{\begin{array}{c} \mu_{t}=\mu_{0}+\mu_{HEAT}{HEAT}_{t}+\mu_{COLD}{COLD}_{t}+\mu_{NO2}{NO2}_{t}+\mu_{O3}{O3}_{t}+\mu_{PM25}{PM25}_{t}+\mu_{flu}p_{flu,t}+\mu_{COVID-19}p_{COVID-19,t}+\mu_{PSCORE\_LAG1}Y_{t-1}\\ \sigma_{t}={exp(\sigma }_{0}+\sigma_{HEAT}{HEAT}_{t}+\sigma_{COLD}{COLD}_{t}+\sigma_{NO2}{NO2}_{t}+\sigma_{O3}{O3}_{t}+\sigma_{PM25}{PM25}_{t}+\sigma_{flu}p_{flu,t}+\sigma_{COVID-19}p_{COVID-19,t}+\sigma_{PSCORE\_LAG1}Y_{t-1}) \end{array}\right. \end{array}$$

*μ_j_* measures the expected change in the mean p-score per unit increase in the covariate *X_j_*, while *σ_j_* captures the change in the dispersion of p-scores.

To identify the most relevant covariates for both *μ_t_* and *σ_t_*, we conducted stepwise selection based on the Hannan–Quinn Information Criterion (HQC) [50]. The process was implemented using the *stepGAICAll.A* function from the R package GAMLSS, specifically designed for distributional regression models. This method was evaluated by [51], who demonstrated its effectiveness in identifying influential predictors within the GAMLSS framework. Model adequacy was examined through normalized quantile residuals [52] and a de-trended QQ plot, also known as a worm plot [53], highlighting any deviations from the assumed logistic distribution.

Sensitivity analyses were performed to evaluate the robustness of the model. These included testing the inclusion of seasonal and long-term trends within the logistic distribution for p-scores, assessing linear time interactions with covariates, and excluding the 2003 heatwave to examine the stability of the model under extreme environmental conditions.

Excess mortality attributable to environmental factors can be quantified using the estimated parameters of the logistic distribution. The expected value of the p-score distribution corresponds to the location parameter *μ*, which enabled us to estimate the contribution of each covariate *Xj* on excess mortality during week *t* as *μ_j_X_t,j_*. Based on this, the number of deaths attributable to the covariate *Xj* for a given year *k* was derived using the following formula, where *n_t_* represents the observed number of deaths in week *t*:

(3) $$\begin{array}{c} \;N_{attributable,k}\left(X_{j}\right)=\sum_{t\;\in \;year\;k}\frac{{n_{t}\mu }_{j}X_{t,j}}{100}\; \end{array}$$

In addition to the number of attributable deaths, the ASMR attributable to the covariate *Xj* for year *k* can be calculated using the baseline ASMR for week *t*, *B_t_*_,_:

(4) $$\begin{array}{c} \;\tau_{attributable,k}\left(X_{j}\right)=\sum_{t\;\in \;year\;k}\frac{{B_{t}\mu }_{j}X_{t,j}}{100}\; \end{array}$$

While Equation (3) quantifies the absolute burden in terms of lives lost, Equation (4) provides a standardized metric that facilitates comparisons across different populations or geographical regions.

Next, we evaluated the ability of the logistic distribution to capture both the occurrence and intensity of extreme excess mortality events. Extreme events were defined as weeks with p-scores exceeding their empirical 90th percentile (*u*_90%_). This threshold was selected in line with the definition of mortality peaks provided in [23]. The probability of an extreme excess mortality event occurring in week *t*, conditionally on values of the covariate *Xj*, was estimated using the logistic distribution. Letting *Y_t_* denote the p-score observed in week *t*, this probability can be expressed as follows:

(5) $$\begin{array}{c} \;p_{t,j}(x>)=P\left(Y_{t}>u_{90\%}\;\right|{\;X}_{t,j}=x)=1-F\left(u_{90\%}\;|\;X_{t,j}=x\right) \end{array}$$

where all other covariates are held at zero. This conditional probability quantifies how the covariate *Xj* influences the likelihood of an extreme p-score. In the case where the scale parameter *σ_t_* does not depend on *Xj*, the odds ratio (OR) for an extreme event occurring due to a one-unit increase in covariate *Xj* can be derived from Equations (1) and (5) as ${OR}_{j}=\exp\left(\mu_{j}/\sigma_{0}\right)$. Note that the OR does not depend on the predefined threshold of u_90%_ in this model: the threshold affects the baseline probability of an event; however, as a characteristic of the logistic distribution, it does not impact the relative increase in odds per unit increase in *Xj*. For comparison, a traditional binomial logistic regression model was also applied to explain the occurrence of extreme excess mortality events, treating the presence of an extreme event as a binary outcome. Explanatory variables were selected through a stepwise backward elimination process, minimizing the HQC, similar to the approach used for the logistic distribution model.

In addition to the occurrence of extremes, the intensity of extreme p-scores was assessed through return levels derived from the logistic distribution. The *T*-year return level *y_T_* corresponds to the p-score having an annual exceedance probability of 1/*T*, verifying $P\left(Y_{max}>y_{T}\right)=1/T$, where $Y_{max}$ denotes annual maximum p-scores, i.e., the highest observed p-score for a given year [54]. *y_T_* is thus expected to be exceeded on average once every *T* years. Given the potential clustering of extreme p-scores across weeks, we incorporated the extremal index θ, which adjusts for temporal dependence in extremes by approximating the annual maximum distribution using the weekly p-score distribution [55]. This leads to $P\left(Y_{max}\leq y\right)\approx F^{\lambda \theta }\left(y\right)$, where *λ* represents the average number of observations per year (365.25/7 for weekly data). We estimated *θ* using the intervals estimator of Ferro and Segers [56] from exceedances above the threshold of *u*_90%_. *y_T_* thus satisfies the following equation:

(6) $$\begin{array}{c} \;y_{T}=F^{-1}\left({\left(1-1/T\right)}^{\frac{1}{\lambda \theta }}\right)=\mu +\sigma log\left(\frac{p}{1-p}\right) \end{array}$$

where ${p=\left(1-1/T\right)}^{\frac{1}{\lambda \theta }}$. To obtain the unconditional return level *y_T_*, we first predicted *μ* and *σ* from observed weekly covariate combinations, as these parameters vary depending on the weekly environmental conditions. We then averaged the conditional quantiles of order *p* across these covariate combinations to derive a return level independent of the covariates [57]. This averaging approach integrates the variability of covariates over the sample period, producing return levels that reflect the full range of covariate values over time.

Return periods were also estimated for excess mortality observed during notable events, specifically the 2003 heatwave and the COVID-19 pandemic. Letting *y_event_* denote the highest p-score observed during the event, the corresponding return period is calculated as follows:

(7) $$\begin{array}{c} \;T=\frac{1}{1-F^{\lambda \theta }\left(y_{event}\right)} \end{array}$$

These return periods represent the likelihood of observing an extreme p-score of similar magnitude and do not imply the recurrence of a heatwave or pandemic event itself.

We compared these return levels and return periods to those estimated using the exponential distribution, a standard model for threshold exceedances in extreme value theory [58]. This distribution was fitted directly to p-scores above the u_90%_ threshold, adjusting for temporal dependence using the extremal index.

Confidence intervals for indicators related to extreme events were estimated through bootstrapping, where weeks were resampled with replacement from the original dataset, and the parameters of the logistic distribution, binomial regression, and exponential distribution were re-estimated.

## 3. Results

### 3.1. Occurrence of Environmental Episodes

**Figure 2:**
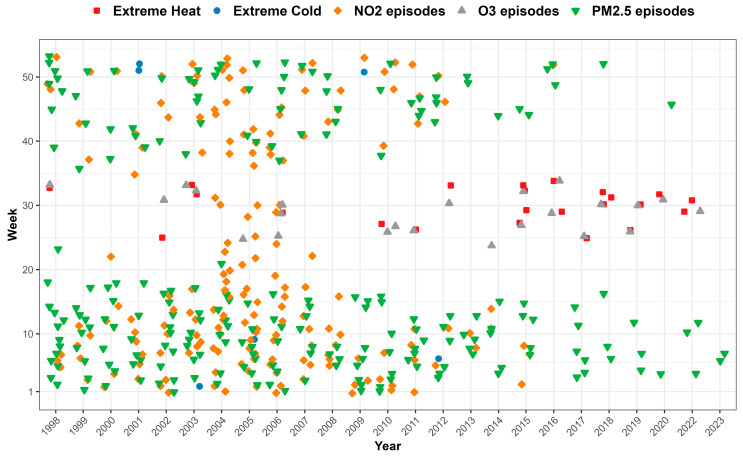
Occurrence of environmental episodes by year and week in Luxembourg from 1998 to 2023.

After applying bias correction to the temperature and pollutant concentration datasets, we constructed a daily national exposure series, with identified environmental episodes based on threshold exceedances defined in Table 1. Figure 2 displays the occurrence of environmental episodes across different years and weeks in Luxembourg. Seasonal and long-term trends are apparent: extreme cold episodes (averaging 0.3 days per year) are concentrated in winter, while PM_2.5_ episodes (18.6 days per year) generally occur in both winter and spring. NO_2_ episodes (11.9 days per year) are distributed throughout the year. Heat episodes (1.8 days per year) and ozone episodes (1.4 days per year) predominantly occur during summer, with a notable pattern of simultaneous extreme heat and ozone events. Additionally, while the frequency of extreme cold, PM_2.5_, and NO_2_ episodes has decreased over time—with no NO_2_ episodes since 2017 and no extreme cold days since 2012—the frequency of extreme heat episodes has increased over the past decade.

### 3.2. Descriptive Analysis of Excess Mortality

**Figure 3:**
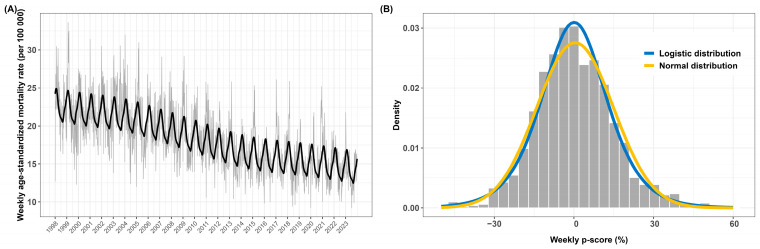
(**A**) Time series of weekly age-standardized mortality rates (ASMRs) per 100,000 population for natural-cause deaths in Luxembourg from 1998 to 2023. The black line represents the baseline mortality derived from median regression. (**B**) Histogram of weekly p-scores overlaid with fitted densities from the normal (yellow) and logistic (blue) distributions.

Panel A in Figure 3 illustrates the weekly ASMRs (per 100,000 population) for natural-cause deaths from 1998 to 2023. The chart also includes the estimated baseline mortality, calculated through median regression, which captures a decreasing long-term trend and a seasonal pattern of increased mortality in winter.

Table 2 provides descriptive statistics of weekly p-scores. The Pearson correlation between weekly p-scores and death counts is 0.81, indicating that high p-scores typically correspond with increased numbers of deaths. The highest excess mortality was recorded in week 50 of 2020, with a p-score of 57.8%. This peak coincided with the height of the COVID-19 pandemic, during which 40 deaths were attributed to the virus, accounting for 32.5% of total deaths for that week. The ASMR rose sharply to 25.21 deaths per 100,000, significantly exceeding the baseline rate of 15.98 deaths per 100,000. No cold or pollution episodes were observed during this week. The second highest p-score, at 55.8%, occurred during the 2003 heatwave in week 32, marked by seven days of extreme heat and two days of ozone episodes. The ASMR during this week reached 30.57 deaths per 100,000, compared to the baseline rate of 19.61 deaths per 100,000.

### 3.3. Statistical Modeling of Excess Mortality

Panel B in Figure 3 displays the histogram of weekly p-scores alongside fitted densities from the normal and logistic distributions. This comparison shows that the normal distribution underestimates both the central values and extremes in the p-score distribution, while the logistic distribution provides a closer fit across the full range of values.

**Figure 4:**
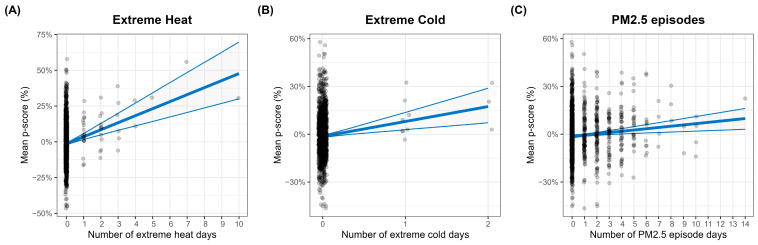
Marginal effects of (**A**) extreme heat days, (**B**) extreme cold days, and (**C**) PM_2.5_ episode days on mean p-scores, based on the *μ* coefficients of the logistic distribution (with 95% CI). Effects are computed with all other covariates set to zero. Dots represent observed p-scores.

The variable selection process for the logistic distribution parameters, based on HQC, identified significant covariates linked with p-scores: extreme heat and cold, PM_2.5_ and NO_2_ episodes, the proportion of COVID-19 deaths, and the lagged p-score from the previous week. A summary of the estimated parameters is presented in Table 3. Extreme heat episodes are associated with higher p-scores, reflected in an estimated parameter μ_HEAT_ = 4.91 (95% CI: [3.15, 6.67]). Extreme cold also emerged as a critical factor, with a parameter estimate of μ_COLD_ = 9.37 (95% CI: [3.64, 15.10]). Additionally, PM_2.5_ episodes resulted in an estimated μ_PM2.5_ = 0.80 (95% CI: [0.29, 1.31]), while the percentage of COVID-19 deaths was associated with μ_COVID-19_ = 0.55 (95% CI: [0.30, 0.80]). The lagged p-score from the previous week also influenced the current p-score, yielding an estimated μ_PSCORE_LAG1_ = 0.08 (95% CI: [0.29; 1.31]). Figure 4 depicts how the mean excess mortality evolves with these covariates. The scale parameter estimates revealed increased variability of excess mortality during NO_2_ episodes and the COVID-19 pandemic, with σ_NO2_ = 0.05 (95% CI: [0.01, 0.09]) and σ_COVID-19_ = 0.02 (95% CI: [0.01, 0.03]).

**Figure 5:**
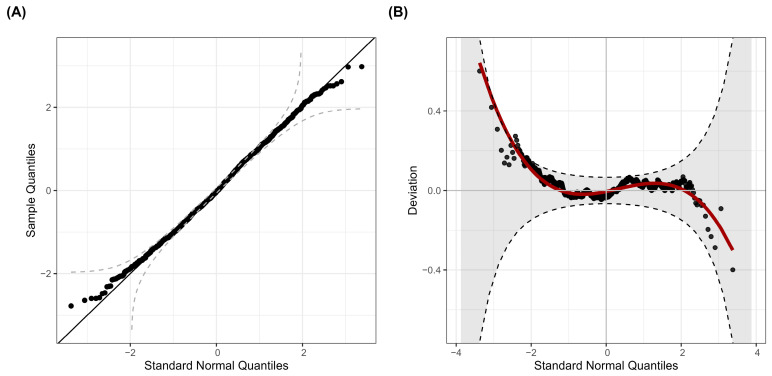
Diagnostics of logistic distribution fit for modeling p-scores. (**A**) Q-Q plot of normalized quantile residuals; (**B**) worm plot highlighting deviation from normality.

The logistic distribution’s goodness of fit was assessed with diagnostic tests shown in Figure 5, which indicated no significant deviation from the logistic distribution. This was further supported by the Kolmogorov–Smirnov test for the normality of the quantile residuals, which yielded a *p*-value of 0.53, hence not rejecting the adequacy of the logistic distribution to model p-scores with the selected covariates.

### 3.4. Attributable Mortality Due to Environmental Episodes

Table 4 summarizes the estimated mortality in Luxembourg attributable to environmental episodes from 1998 to 2023, with a specific focus on the most recent period (2019–2023). Over 1998–2023, extreme heat is associated with an estimated 12.59 deaths per year on average (95% CI: [8.07, 17.11]) and a corresponding annual ASMR of 2.82 deaths per 100,000 population (95% CI: [1.81, 3.83]). Extreme cold contributes to approximately 3.95 deaths per year (95% CI: [1.53, 6.36]), with an annual ASMR of 1.12 deaths per 100,000 population (95% CI: [0.43, 1.80]). The highest impact is observed with PM_2.5_ episodes, which are estimated to cause 21.16 deaths per year (95% CI: [7.75, 34.57]) and an annual ASMR of 6.32 deaths per 100,000 population (95% CI: [2.32, 10.33]). However, both indicators have shown a notable decline in the most recent period (2019–2023), attributed to the reduced frequency of PM_2.5_ episodes, which decreased from an average of 18.58 episodes per year over 1998–2023 to 3.00 episodes per year during 2019–2023.

**Figure 6:**
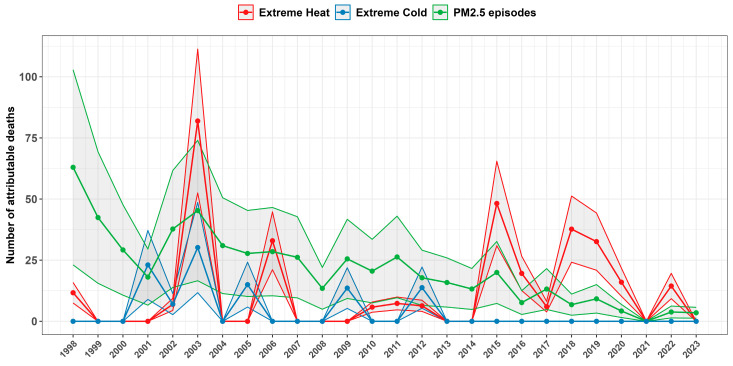
Yearly time series of the number of natural deaths attributed to environmental episodes in Luxembourg from 1998 to 2023.

Figure 6 shows the variation in yearly number of deaths attributed to these episodes, with a peak in 2003 during the 10-day heatwave, resulting in 82 deaths due to extreme heat (95% CI: [52, 111]).

### 3.5. Occurrence and Intensity of Extreme Excess Mortality

The logistic distribution’s capacity to explain the occurrence of extreme excess mortality events, defined as the top 10% of p-scores (i.e., p-scores exceeding u_90%_ = 18%), was evaluated alongside a binomial logistic regression model. In the binomial model, a backward selection process using the HQC criterion identified five key predictors for extreme events: extreme heat, extreme cold, PM_2.5_ episodes, the proportion of COVID-19-related deaths, and the p-score from the previous week.

The ORs for each predictor, shown in Table 5, indicate that both models associate an increased likelihood of extreme mortality with changes in the corresponding environmental variables. Specifically, the OR for extreme heat was 1.93 (95% CI: [1.52, 2.66]) in the logistic distribution, slightly lower than the binomial regression’s OR of 1.98 (95% CI: [1.45, 2.71]). For extreme cold, the logistic distribution showed an OR of 3.49 (95% CI: [1.77, 7.56]) compared to 4.39 (95% CI: [1.73, 11.16]) in the binomial regression. ORs for PM_2.5_ episodes were nearly identical between models, with 1.11 (95% CI: [1.04, 1.19]) in the logistic distribution and 1.12 (95% CI: [1.01, 1.24]) in the binomial regression.

**Figure 7:**
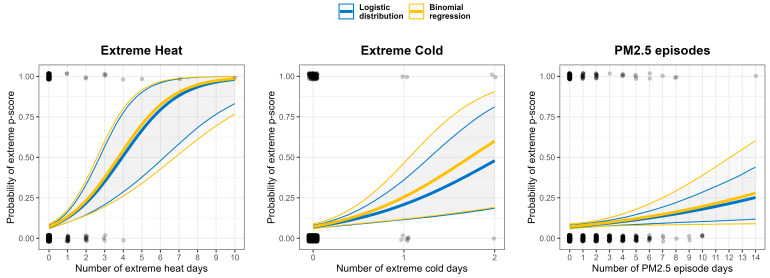
The probability of extreme excess mortality occurrence under environmental risks, estimated from logistic distribution (blue) and binomial regression (yellow). Dots indicate if extreme mortality occurred in the sample.

Figure 7 presents the probability of extreme excess mortality events as a function of these covariates. Both models indicate a probability of ≥50% for extreme p-scores from four days of extreme heat or two days of extreme cold and approximately 25% after two full weeks of PM_2.5_ episodes. The logistic distribution model produced estimates closely aligned with those from the binomial regression, though with reduced variability, indicating a more stable model fit.

**Figure 8:**
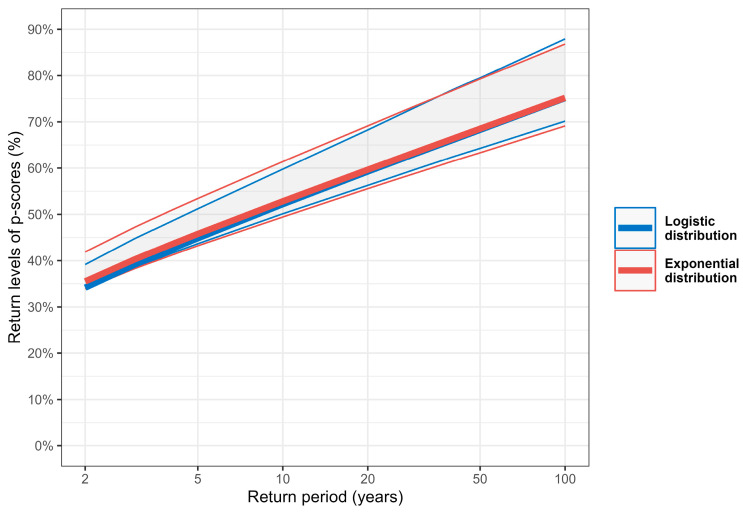
Return level plot of weekly p-scores, comparing estimates from the logistic (blue) and exponential (red) distributions, with 95% confidence intervals.

Concerning the intensity of extreme excess mortality events, we compared return levels estimated using Equation (6) with those from an exponential distribution directly fitted to exceedances above the threshold of u_90%_. Figure 8 and Table 6 show the estimated return levels, highlighting a strong alignment between the two models. For instance, the 50-year return level, i.e., the p-score expected to be exceeded on average once every 50 years, is estimated at 68.3% under the logistic model (95% CI: [64.3%, 79.5%]) and at 68.5% under the exponential model (95% CI: [63.3%, 79.3%]). The extremal index, used to adjust for the temporal clustering of extremes, was estimated as *θ* = 0.76, indicating that p-score exceedances above u_90%_ tend to persist for an average duration of 1/*θ* = 1.32 weeks.

Return periods for the highest observed p-scores during the COVID-19 pandemic and the 2003 heatwave are provided in Table 7. During the COVID-19 pandemic, the p-score in week 50 of 2020 (57.8%) corresponds to a return period of approximately 17.3 years under the logistic model (95% CI: [8.5, 23.8 years]) and 16.6 years under the exponential model (95% CI: [7.6, 26.8 years]). The p-score observed during the 2003 heatwave in week 32 (55.8%) has an estimated return period of 14.3 years according to the logistic model (95% CI: [7.2, 19.1 years]) and 13.6 years for the exponential model (95% CI: [6.4, 21.2 years]).

## 4. Discussion

These results support existing evidence on the short-term impact of extreme temperatures and PM_2.5_ pollution on excess mortality in Luxembourg.

Although extreme cold events cause a larger immediate increase in mean p-scores (9.4%) compared to extreme heat (4.9%), their relative rarity implies that cumulative mortality due to extreme heat remains higher, with 12.6 deaths annually compared to 3.95 for cold. The frequency of extreme heat days appears stable (1.77 per year on average in 1998–2023; 1.80 per year in 2019–2023). If the 2003 heatwave is excluded, this rate lowers to 1.44 during the period 1998–2023, underscoring both the singular severity of that event and a potential upward trend in extreme heat frequency. This trend, exacerbated by climate change, suggests increasing future heat-related mortality [59,60].

In this analysis, the variable selection process identified NO_2_ episodes as influencing the variability (rather than the average) of excess mortality, suggesting that short-term spikes in NO_2_ levels lead to fluctuations in weekly p-scores rather than systematically increasing their mean values. When PM_2.5_ is excluded from the model, NO_2_ appears in the mean (*μ*_NO2_ = 0.94, 95% CI = [0.16, 1.71]), suggesting a confounding relationship between NO_2_ and PM_2.5_ for mortality outcomes. Similar findings have been observed in other studies: NO_2_ significantly affects mortality in single-pollutant models, but its impact often diminishes in multi-pollutant models after adjusting for PM_2.5_ [61,62,63]. Another confounding effect was observed between ozone and extreme heat, as 70% of ozone episodes coincided with extreme heat days, leading to the exclusion of ozone episodes from the model. When extreme heat is not considered, ozone episodes become significant with an estimated effect of *μ*_O3_ = 3.81 (95% CI = [1.78, 5.85]). This finding aligns with [64], which indicates that ozone’s mortality impact is primarily heat-driven: ozone levels rise with high temperatures, posing an additional health risk that intensifies mortality during heatwaves. Testing alternative ozone thresholds (e.g., 120 µg/m^3^) produced identical conclusions.

Air quality improvements are reflected in the declining frequency of pollution episodes over time. While PM_2.5_ episodes had the highest mortality impact over the entire period, Figure 6 shows a decreasing trend in PM_2.5_-related deaths, as episode days dropped from an average of 18.6 per year (1998–2023) to 3.0 per year (2019–2023). Additionally, NO_2_ episodes have declined from 11.9 days annually over the full period, with no episodes recorded from 2019 to 2023, consistent with air quality improvements documented in previous studies [65,66].

Sensitivity analyses were conducted to assess the influence of selected covariates in the model. Seasonal and long-term trends were tested within the logistic distribution for p-scores but were ultimately excluded during the variable selection process. This indicates that these variations were adequately captured by the baseline mortality, making further temporal adjustments unnecessary for modeling p-scores. Similarly, testing for linear time interactions with covariates revealed no significant temporal changes in the effects of environmental factors. The impact of extreme heat on mortality remained consistent even when excluding the 2003 heatwave (*μ_HEAT_* = 4.91, 95% CI: [3.15, 6.67] overall; *μ_HEAT_* = 4.96, 95% CI: [2.98, 6.94] without the 2003 heatwave), highlighting the model’s robustness against extreme environmental events.

Our findings also indicate a short-term memory effect in weekly excess mortality, reflected by the autoregressive component selected in the logistic distribution (*μ_PSCORE_LAG1_* = 0.08, 95% CI = [0.03, 0.13]). This supports previous modeling approaches that included autoregressive processes to capture mortality dynamics [67,68,69]. Moreover, the clustering of extreme mortality events, evidenced by an average duration of approximately 1.32 weeks, points to the persistence of mortality effects following shocks. This clustering is particularly relevant for healthcare capacity planning, as periods with high excess mortality are likely to exert increased pressure on healthcare systems.

The logistic distribution effectively captured both the probability and intensity of extreme excess mortality, aligning well with models specifically designed for extreme events. In particular, return levels and return periods contextualize the severity of recent and historical extreme events, with return periods estimated at 17.3 years for the COVID-19 peak p-score (95% CI: [8.5, 23.8 years]) and 14.3 years for the 2003 heatwave p-score (95% CI: [7.2, 19.1 years]). Although widely used in fields such as hydrology, coastal engineering, or finance, these measures could also serve as benchmarks in public health to enhance preparedness for severe mortality events.

This study provides key insights for public health authorities and policymakers in mitigating environmental health risks. The findings, including attributable ASMR estimates (averaging 2.8 deaths per 100,000/year for extreme heat, 1.1 for extreme cold, and 6.3 for PM_2.5_ episodes), underscore the need for targeted interventions. The observed decline in PM_2.5_-related deaths reflects the effectiveness of air quality improvements, while persistent heat-related risks highlight the urgency of enhanced preparedness measures, such as early warning systems and adaptive healthcare strategies. The identification of confounding effects between NO_2_ and PM_2.5_, as well as between ozone and extreme heat, also reinforces the need for integrated climate and air quality policies. Moreover, excess mortality p-score return levels could serve as benchmarks to refine preparedness planning and optimize healthcare resource allocation.

This study has some limitations. First, as Luxembourg is a small country with relatively few death cases, we opted for a weekly rather than daily analysis to reduce potential noise introduced by a daily approach. However, this temporal resolution may obscure finer, shorter-term associations between environmental risks and excess mortality. Additionally, the reliance on aggregated mortality data rather than individual-level analysis might introduce unmeasured confounding factors. Furthermore, as this study relies on age-standardized mortality rates, it provides a broad view of population-wide impacts but does not capture age-specific variations. The literature shows that environmental risks disproportionately affect older populations [70,71], potentially leading to an underestimation of the risks faced by elderly individuals. Moreover, defining environmental risks with threshold-based events clarified extreme conditions but ignored associations at lower exposures. A continuous variable approach could better capture the full exposure spectrum.

Lastly, the definitions of extreme heat, cold, and air pollution episodes were chosen for policy relevance, using absolute thresholds aligned with Luxembourg’s national alert system and EU 2030 air quality standards. While this approach enhances direct applicability to national and regulatory frameworks, it may limit comparability with studies using different or percentile-based thresholds.

Further research could address several areas to expand on our findings. Incorporating geographic units, such as the cantonal level, could enhance the models and provide more localized results. Stratification by age would also facilitate the identification of specific age groups most vulnerable to environmental risks, enabling more targeted public health strategies. Future work could also explore the impact of using absolute versus relative thresholds for defining environmental episodes and balancing policy relevance with climatological adaptability. Moreover, following the approach of [72], the estimated models could be integrated into an operational real-time system for forecasting weekly environmental-related excess mortality in Luxembourg.

## 5. Conclusions

This study highlights the impact of extreme temperatures and air pollution episodes on excess mortality from natural causes in Luxembourg. Extreme heat, cold, and PM_2.5_ pollution episodes were major contributors, with confounding effects identified between ozone and heat, as well as between NO_2_ and PM_2.5_ episodes. The originality of this work resides in the application of a distributional regression model, which allowed us to assess not only average impacts but also variability in p-scores, along with the occurrence and intensity of extreme mortality events. Key indicators derived from the model include attributable deaths, probabilities of extreme mortality, and return periods of excess mortality that occurred during exceptional events like the 2003 heatwave or the COVID-19 pandemic peak. While the analysis focused on Luxembourg, the methods are widely applicable to other regions with different climatology or air quality levels. Future work could rely on these models to develop an operational framework for real-time forecasting of mortality risks based on short-term environmental conditions. Such advancements would enhance public health emergency preparedness and offer a practical tool for mitigating the harmful impacts of extreme environmental episodes on health.

## Figures and Tables

**Table 1:** Definitions and thresholds of the environmental risks analyzed in this study.

Environmental Risk	Definition
Extreme heat	Maximal daily temperature ≥ 35 °C and previous day’s average temperature ≥ 23 °C
Extreme cold	Minimal daily temperature ≤ −15 °C
PM_2.5_ episode	24 h average PM_2.5_ concentration ≥ 25 µg/m^3^
NO_2_ episode	24 h average NO_2_ concentration ≥ 50 µg/m^3^
O_3_ episode	1h daily maximum O_3_ concentration ≥ 160 µg/m^3^

**Table 2:** Descriptive statistics of weekly excess mortality p-scores.

Minimum	1st Quartile	Median	Mean	3rd Quartile	Maximum
−47.6%	−9.2%	−0.5%	0.4%	9.4%	57.8%

**Table 3:** Estimated logistic distribution parameters for weekly p-scores, modeled in relation to environmental risks.

Parameter	Covariate	Estimate	Standard Error	95% CI	*p*-Value
*μ*	(Intercept)	−1.36	0.42	[−2.20, −0.54]	0.00129
	Extreme heat	4.91	0.90	[3.15, 6.67]	5.67 × 10^−8^
	Extreme cold	9.37	2.93	[3.64, 15.10]	0.00139
	PM_2.5_ episodes	0.80	0.26	[0.29, 1.31]	0.00203
	COVID-19	0.55	0.13	[0.30, 0.80]	2.20 × 10^−5^
	PSCORE_LAG1	0.08	0.03	[0.03, 0.13]	0.00169
*σ*	(Intercept)	2.01	0.03	[1.96, 2.06]	<2 × 10^−16^
	NO_2_ episodes	0.05	0.02	[0.01, 0.09]	0.02127
	COVID-19	0.02	0.01	[0.01, 0.03]	0.00497

**Table 4:** Mortality attributable to environmental episodes in Luxembourg from 1998 to 2023, including total and annual estimates.

Environmental Risk	Annual Frequency of Episode Days		Total Attributable Number of Deaths (with 95% CI)		Yearly Average Attributable Number of Deaths (with 95% CI)		Yearly Average Attributable ASMR per 100,000 (with 95% CI)	
	1998–2023	2019–2023	1998–2023	2019–2023	1998–2023	2019–2023	1998–2023	2019–2023
Extreme Heat	1.77	1.80	327 [210, 445]	63 [40, 86]	12.59 [8.07, 17.11]	12.60 [8.08, 17.12]	2.82 [1.81, 3.83]	2.39 [1.53, 3.25]
Extreme Cold	0.27	0.00	103 [40, 165]	0 [0, 0]	3.95 [1.53, 6.36]	0 [0, 0]	1.12 [0.43, 1.80]	0 [0, 0]
PM_2.5_ Episodes	18.58	3.00	550 [201, 899]	21 [8, 34]	21.16 [7.75, 34.57]	4.14 [1.52, 6.77]	6.32 [2.32, 10.33]	0.87 [0.32, 1.42]

**Table 5:** Odds ratios for the occurrence of extreme excess mortality events derived from logistic and binomial regression.

Environmental Risk	Odds Ratio (with 95% CI)	
	Logistic Distribution	Binomial Regression
Extreme Heat	1.93 [1.52, 2.66]	1.98 [1.45, 2.71]
Extreme Cold	3.49 [1.77, 7.56]	4.39 [1.73, 11.16]
PM_2.5_ Episodes	1.11 [1.04, 1.19]	1.12 [1.01, 1.24]

**Table 6:** Return levels of weekly p-scores for specified return periods, estimated using logistic and exponential distributions (with 95% confidence intervals).

Return Period (Years)	Excess Mortality p-Scores Return Levels (%, with 95% CI)	
	Logistic Distribution	Exponential Distribution
5	44.9% [43.7%, 51.2%]	45.8% [43.2%, 53.4%]
10	52.2% [50.1%, 59.8%]	52.8% [49.4%, 61.4%]
25	61.5% [58.2%, 71.1%]	61.8% [57.5%, 71.6%]
50	68.3% [64.3%, 79.5%]	68.5% [63.3%, 79.3%]
100	75.1% [70.2%, 87.9%]	75.2% [69.1%, 86.8%]

**Table 7:** Estimated return periods for the highest weekly p-scores observed during the COVID-19 pandemic and the 2003 heatwave.

Event	Week of Highest p-Score	Highest p-Score (%)	Return Period from Logistic Distribution (years, with 95% CI)	Return Period from Exponential Distribution (Years, with 95% CI)
COVID-19 pandemic	2020-W50	57.8%	17.3 [8.5, 23.8]	16.6 [7.6, 26.8]
2003 heatwave	2003-W32	55.8%	14.3 [7.2, 19.1]	13.6 [6.4, 21.2]

## Data Availability

Access requests for the data (along with the code for this study) can be channeled to the author through jerome.weiss@ms.etat.lu.
